# Supplementary material for: Adult zebra finches rehearse highly variable song patterns during sleep
Source: PeerJ. 2017 Nov 16;5:e4052. doi: 10.7717/peerj.4052 (PMC5694654; doi:10.7717/peerj.4052)
Supplement: Table S1 [file peerj-05-4052-s002.docx]

Table S1: Recorded muscles for each bird.

| Bird | BR192 | G81 | G74 | G51 | P98 | P106 | P145 | P144 | P188 | P181 |
| --- | --- | --- | --- | --- | --- | --- | --- | --- | --- | --- |
| Muscles | dS_L_*, vTB_L_ | dS_L_, vTB_L_ | vTB_L_, vTB_R_ | vTB_L_, vTB_R_ | vS_L_,  vS_R_ | vTB_L_, vS_R_ | dS_R_, vTB_R_ | dS_R_, vTB_R_ | vTB_L_, vTB_R_ | vS_L_,  vS_R_ |

_*L and R indicate left or right muscle._
