# Supplementary material for: Adult zebra finches rehearse highly variable song patterns during sleep
Source: PeerJ. 2017 Nov 16;5:e4052. doi: 10.7717/peerj.4052 (PMC5694654; doi:10.7717/peerj.4052)
Supplement: Table S2 [file peerj-05-4052-s003.docx]

Table S2: Average occurrence of each SLA type with standard error.

| Category | 1 Syllable | Syllable + unknown | Two or more syllables | Two or more + unknown | Complete motifs | Partial syllables | Incorrect timing |
| --- | --- | --- | --- | --- | --- | --- | --- |
| Percent occurrence | 20±6.6% | 12±4.2% | 15±4.3% | 5.5±2.2% | 6.1±2.2% | 33±7.4% | 8.1±2.0% |
